# Review of New Clinical Aspects of Cardiac Pathology in Patients with COVID-19 Infection

**DOI:** 10.15388/Amed.2021.28.1.12

**Published:** 2021-03-09

**Authors:** Audrė Alonderytė, Giedrius Navickas, Robertas Stasys Samalavičius, Pranas Šerpytis

**Affiliations:** Faculty of Medicine, Vilnius University, Vilnius, Lithuania; Centre of Cardiology and Angiology, Vilnius University Hospital Santaros Klinikos, Vilnius, Lithuania; Institute of Clinical Medicine, Faculty of Medicine, Vilnius University, Vilnius, Lithuania Emergency Medicine Center, Vilnius University Hospital Santaros Klinikos, Vilnius, Lithuania; Centre of Cardiology and Angiology, Vilnius University Hospital Santaros Klinikos, Vilnius, Lithuania Institute of Clinical Medicine, Faculty of Medicine, Vilnius University, Vilnius, Lithuania Emergency Medicine Center, Vilnius University Hospital Santaros Klinikos, Vilnius, Lithuania

**Keywords:** coronavirus, acute coronary syndrome, myocardial infarction, treatment

## Abstract

**Summary. Background:**

COVID-19 disease is a huge burden for society and healthcare specialists. As more information is gathered about this new disease, it becomes clear that it affects not only respiratory, but also cardiovascular system.

**Materials and Methods:**

The aim of this review is to analyse the information about myocardial injury caused by COVID-19 and overview treatment options for these patients in publications which were published in the last 5 years. The data for this overview were collected in the PubMed database. Full-text articles were used for analysis when their title, summary, or keywords matched the purpose of the review. Only publications published in English that appeared in the last 5 years were analysed. For the analysis 14 publications were selected and analysed.

**Conclusion:**

COVID-19 infection could mimic ST-elevation myocardial infarction and it is crucial to differentiate the main cause and choose the appropriate treatment. Cardiovascular complications are related with poorer prognosis and higher mortality. This should be thoroughly considered by the healthcare specialists in order to choose appropriate treatment strategy. Patients with acute coronary syndrome (ACS) due to plaque rupture should receive dual antiplatelet therapy and full-dose anticoagulation if it is not contraindicated. Therefore, priority should be given to the acute coronary syndrome given the low evidence of new antiviral treatment effectiveness. Number of agents which are under investigation for COVID-19 may have interactions with oral antiplatelet drugs. Selected patients could receive immunosuppressive treatment as well as extracorporeal membrane oxygenation as a bridge to recovery.

## Abstract (lt)

**Santrauka. Įvadas:**

COVID-19 liga – didžiulė našta visuomenei ir sveikatos priežiūros specialistams. Vis daugėjant duomenų apie pačią ligą ir didėjant atliktų tyrimui skaičiui, matoma tendencija, kad SARS-CoV-2 tiesiogiai paveikia ne tik kvėpavimo sistemą, bet ir širdies ir kraujagyslių sistemą.

**Metodai:**

šios apžvalgos tikslas – išanalizuoti informaciją apie COVID-19 sukeltą miokardo pažeidimą ir apžvelgti jį turinčių pacientų gydymo galimybes, analizuojant ne senesnes nei 5 metų publikacijas. Šios apžvalgos duomenys buvo renkami „PubMed“ duomenų bazėje. Analizei naudoti visateksčiai straipsniai, kurių pavadinimas, santrauka ar raktažodžiai atitiko apžvalgos tikslą. Analizuotos tik anglų kalba skelbtos publikacijos, pasirodžiusios ne vėliau nei prieš 5 metus. Analizei atrinkta ir išanalizuota 14 publikacijų.

**Išvados:**

COVID-19 liga gali imituoti miokardo infarktą su ST-segmento pakilimu, todėl svarbu nustatyti pagrindinę priežastį ir tinkamai pasirinkti gydymo taktiką. Širdies ir kraujagyslių sistemos komplikacijos yra susijusios su prastesne prognoze ir didesniu mirtingumu. Atsižvelgdami į tai, sveikatos priežiūros specialistai turėtų apsvarstyti tinkamiausias pacientų gydymo galimybes. Pacientams, sergantiems ūminiu vainikinių arterijų sindromu, turėtų būti skiriama dviguba antiagregacinė terapija ir antikoaguliacija, jei nėra kontraindikacijų. Kuriami nauji specifiniai COVID-19 ligai gydyti skirti vaistai gali sąveikauti su geriamaisiais antiagregantais, todėl pirmenybė turėtų būti skiriama ūminiam vainikinių arterijų sindromo gydymui, atsižvelgiant į tai, kad kuriamų naujų vaistų veiksmingumas nėra iki galo įrodytas. Reikėtų atkreipti dėmesį ir į tai, kad atskiroms populiacijoms galėtų būti taikomas imunosupresinis gydymas arba ekstrakorporinė membraninė oksigenacija kaip tiltas į pasveikimą.

**Raktažodžiai**: koronavirusas, ūminis vainikinių arterijų sindromas, miokardo infarktas, gydymas.

## Introduction

Severe acute respiratory syndrome corona virus 2 (SARS-CoV- 2) originated in Wuhan, China, in December 2019. This virus causes coronavirus disease 2019 (COVID-19) and is highly contagious and poses a major threat to public health. The vast majority of COVID-19 have had a good prognosis, but there were still some critical individuals and even deaths [1]. Systemic viral infections have been associated with acute myocardial infarction, inflammation and may be a pathophysiologic trigger for plaque rupture and thrombosis. Cardiovascular risk factors or prior cardiovascular diseases are associated with poorer prognosis. These patients are more likely to experience severe or critical COVID-19 illness requiring intensive care unit (ICU) use for advanced therapies including vasopressors for hemodynamic support, mechanical ventilation, and mechanical circulatory support including extracorporeal membrane oxygenation (ECMO) [2].

## Discussion

The most severe clinical manifestations for these critically ill and dead patients are developed in the later stages of the disease or the process of recovery. Cytokine storm is considered to be responsible for disease aggravation and multiple organ failure [1].

Acute thrombosis, myocarditis, myocardial ischemia are reported in 7-28% of COVID-19 positive patients and this is related with higher mortality [2]. In comparison with previous coronavirus outbreaks, COVID-19 presents with a higher incidence of cardiovascular complications [3]. It is believed that myocardial damage could be caused by several mechanisms: hyperinflammation and cytokine storm leading to myocarditis, respiratory failure and hypoxemia causing damage to cardiomyocytes, downregulation of ACE2 expression, hypercoagulability which leads to coronary microvascular thrombosis, diffuse endothelial injury, inflammation and stress-causing coronary plaque rupture or supply–demand mismatch leading to myocardial ischemia/infarction [4]. Santoso et al. performed meta-analysis which demonstrated that acute cardiac injury, represented by elevated troponin concentration was related with increased mortality, the need for ICU use, and severe COVID-19 [5]. In addition to this, COVID-19 patients, who present with cardiac injury (troponin >99^th^ percentile upper reference limit) have 4 times increased risk of mortality. The prognosis is worse in patients with underlying cardiovascular disease [3]. The incidence of myocardial injury in COVID-19 infection tends to be higher. Hence, this may be associated with the lack of standard definition as it is primarily based on elevated serum levels of cardiac-specific troponins. These biomarkers can also be elevated in noncardiac conditions, such as sepsis or critical illness [6]. Hs-troponin should be measured at the time of hospitalization with longitudinal monitoring and should be reviewed in conjunction with other markers of inflammation (e.g. ferritin, IL-6, etc.) in order to distinguish underlying etiology [4]. Patients with heart failure on left ventricular assist device (LVAD) support should be considered as a risk population for COVID-19 infection. Inflammatory and myocardial injury biomarkers should be differentiated with baseline inflammation, which occurs in LVAD patients. Changes in the pattern of these laboratory markers may be useful to follow LVAD with COVID-19 infection [7].

More importantly, electrocardiogram (ECG) changes can mimic an acute myocardial infarction [6]. COVID-19 positive patients, who presented with ST-segment elevation on ECG showed various findings. Angiography revealed obstructive coronary artery disease (CAD), nonobstructive CAD, normal coronary arteries, or left ventricular dysfunction due to myocarditis or stress-induced cardiomyopathy [2]. Wall motion abnormalities (WMA) and myocardial perfusion could be monitored with focused perfusion echocardiography (FPE) as it can differentiate between coronary perfusion distribution patterns. Definite or probable regional WMA indicates myocardial infarction (MI) and other WMA pattern and perfusion abnormalities could be helpful to further differentiate myocarditis, thrombotic microangiopathy, or stress cardiomyopathy. If MI is suspected, it should be differentiated between type 1 and 2 MI. ST-segment elevation myocardial infarction (STEMI) with non-severe COVID-19 disease and unstable non-ST-segment elevation myocardial infarction (NSTEMI) with nonsevere COVID-19 disease should be treated with an early invasive strategy, provided that benefit from revascularization exceeds the risks. However, STEMI can be considered for thrombolysis after evaluating the bleeding risk, age in patients with severe illness, or anticipated delays in percutaneous reperfusion [8]. However, physicians treating STEMI patients with COVID-19 in China recommend fibrinolytic therapy over primary percutaneous coronary intervention (PCI) in stable patients who present within 12 hours of symptom onset and do not have contraindication for fibrinolytics [9]. Due to the improvement of PCI techniques, advances in coronary stent design and effective antithrombotic therapies, coronary stent thrombosis became a rare complication [10]. Hamadeh et al. assessed in the study that patients who were treated with primary PCI had a 21% rate of stent thrombosis, which is much higher than previously reported rates of early stent thrombosis of 1% [9]. It is crucial to weight the benefit/risk ratio for PCI. Patients with short ischemic time, less relative myocardium (e.g. inferior wall) should be treated with fibrinolytic agent. However, the invasive strategy is strongly indicated for the STEMI patients who are older, with longer ischemic time, have massive myocardial injury, or conservative reperfusion strategy was unsuccessful [11]. In Spain, PCI procedures have been reduced by 48%, with a reduction of 40% for primary angioplasty [10], [12]. Additionally, admissions for STEMI have decreased during the COVID-19 pandemic, and reasons for this remained unclear. It may be that patients are not reaching out to the hospital, what potentially leads to the need for mechanical circulatory support in the acute setting, mechanical complications of STEMI, sudden death, or an increased heart failure population [2].

Cytokine storm promotes the coagulation cascade and platelet activation triggered by IL-6 and tissue factor [10]. Mild thrombocytopenia and increased D-dimer levels are the most common findings in COVID-19 positive patients and are related to severe disease or death [13]. It was assessed that 71% of COVID-19 patients who have died met the International Society on Thrombosis and Hemostasis (ISTH) criteria for disseminated intravascular coagulation (DIC) [13]. Number of agents which are under investigation for COVID-19 may have interactions with oral antiplatelet drugs. It should be noted, that drugs metabolized through CYP3A4 (clopidogrel, ticagrelor) could interact with antiviral agents (lopinavir, ritonavir) depending on their effect on metabolism [10], [13]. Patients with acute coronary syndrome (ACS) due to plaque rupture should receive dual antiplatelet therapy and full-dose anticoagulation if it is not contraindicated [13]. Antiplatelet therapy should be prioritized for COVID-19 patients with ACS because of low evidence of the antiviral agents effectiveness [10]. Nonurgent cardiac procedures should be postponed and usual optimal medical therapy should be continued to minimize exposure for patients and health care workers [13].

It could be useful to check for hyperinflammation in severe COVID-19 patients using laboratory markers (e.g. increasing ferritin, decreasing platelet counts, erythrocyte sedimentation rate), and the HScore to identify the patients for whom immunosuppression could be favorable. This could include steroids, intravenous immunoglobulin, selective cytokine blockade (e.g. tocilizumab) and Janus kinase inhibition [14]. In addition to this, VA-ECMO could be reserved for selected cases of COVID-19 patients who suffer from cardiogenic shock and have echocardiographic evidence of reduced biventricular function as a bridge to recovery [3]. 

## Conclusion

COVID-19 can manifest not only with acute respiratory distress syndrome and multiple organ failure but also with acute cardiac injury. Attention should be paid to these patients because poorer outcomes are associated with elevated troponin concentration. COVID-19 infection could mimic ST-elevation myocardial infarction and it is crucial to differentiate the main cause and choose the appropriate treatment. It should be noted, that antiviral treatment could have drug-drug interactions. Selected patients could receive immunosuppressive treatment or VA-ECMO as a bridge to recovery.
